# A Bayesian localized conditional autoregressive model for estimating the health effects of air pollution

**DOI:** 10.1111/biom.12156

**Published:** 2014-02-24

**Authors:** Duncan Lee, Alastair Rushworth, Sujit K Sahu

**Affiliations:** 1 School of Mathematics and Statistics, University of GlasgowGlasgow G12 8QW, UK; 2 Southampton Statistical Sciences Research Institute, University of SouthamptonSouthampton SO17 1BJ, UK

**Keywords:** Air pollution and health, Conditional autoregressive models, Spatial autocorrelation

## Abstract

Estimation of the long-term health effects of air pollution is a challenging task, especially when modeling spatial small-area disease incidence data in an ecological study design. The challenge comes from the unobserved underlying spatial autocorrelation structure in these data, which is accounted for using random effects modeled by a globally smooth conditional autoregressive model. These smooth random effects confound the effects of air pollution, which are also globally smooth. To avoid this collinearity a Bayesian localized conditional autoregressive model is developed for the random effects. This localized model is flexible spatially, in the sense that it is not only able to model areas of spatial smoothness, but also it is able to capture step changes in the random effects surface. This methodological development allows us to improve the estimation performance of the covariate effects, compared to using traditional conditional auto-regressive models. These results are established using a simulation study, and are then illustrated with our motivating study on air pollution and respiratory ill health in Greater Glasgow, Scotland in 2011. The model shows substantial health effects of particulate matter air pollution and nitrogen dioxide, whose effects have been consistently attenuated by the currently available globally smooth models.

## 1. Introduction

Quantification of the health effects of air pollution is an important problem of considerable public interest, both in terms of its financial and health impact. In the UK, the Department for the Environment, Food and Rural Affairs (DEFRA) estimate that “in 2008 air pollution in the form of anthropogenic particulate matter (PM) alone was estimated to reduce average life expectancy in the UK by 6 months. Thereby imposing an estimated equivalent health cost of £19 billion” (DEFRA Air Quality Subject Group, 2010). These estimates are based on large numbers of epidemiological studies, which have quantified the impact of both short-term and long-term exposure. The effects of long-term exposure can be estimated from individual-level cohort studies such as Laden et al. (2006) and Beverland et al. (2012), but they are expensive and time consuming. Therefore ecological small-area study designs have also been used, including Elliott et al. (2007), Lee, Ferguson, and Mitchell (2009), Haining et al. (2010), and Greven, Dominici, and Zeger (2011). While these studies cannot assess the causal health effects of air pollution due to their ecological design, they are quick and cheap to implement, and they contribute to, and independently corroborate, the body of evidence about the long-term population level impact of air pollution.

This ecological design is a form of geographical association study, where the study region is partitioned into non-overlapping areal units, such as counties or census tracts. The number of disease cases observed in each areal unit is modeled, using Poisson regression, by risk factors including air pollution concentrations, socio-economic deprivation, and demography. However, residual spatial autocorrelation may remain in these data, due to unmeasured confounding, neighborhood effects (where individual areal unit's behavior is influenced by that of neighboring units) and grouping effects (where individual units seem to be close to similar units). This autocorrelation is accounted for by adding a set of random effects to the model, which are usually represented by a conditional autoregressive (CAR; Besag, York, and Mollie, 1991) prior as part of a hierarchical Bayesian model.

The majority of CAR priors are globally smooth, and have recently been shown by Reich, Hodges, and Zadnik (2006), Hodges and Reich (2010), Paciorek (2010), and Hughes and Haran (2013) to be potentially collinear with any covariate such as air pollution that is also globally smooth. Such collinearity leads to poor estimation performance for the fixed effects, and additionally suggests that the residual spatial autocorrelation is unlikely to be globally spatially smooth as that component of the spatial variation in the disease data will have been accounted for. Instead, the residual spatial autocorrelation is likely to be strong in some areas showing smoothness, and weak in some other areas exhibiting abrupt step changes. The widely used intrinsic and convolution CAR models proposed by Besag et al. (1991) force the random effects to exhibit a single global level of spatial smoothness determined by geographical adjacency, and are thus not flexible enough to capture the complex localized structure likely to be present in the residual spatial autocorrelation. The lack of flexibility in the intrinsic and convolution CAR models and the collinearity problems highlighted by Hodges and Reich (2010) and others has motivated us to develop a new localized conditional autoregressive (LCAR) prior for modeling residual spatial autocorrelation, which is presented in Section 3. Existing solutions to these problems have been proposed by Reich et al. (2006), Hughes and Haran (2013), and Lee and Mitchell (2013), and a selection of them are compared by simulation to the LCAR prior proposed in this article in Section  4.

To contain the required flexibility, the LCAR prior captures localized residual spatial autocorrelation by allowing random effects in geographically adjacent areas to be autocorrelated or conditionally independent, and we show that this prior distribution can have realizations at both spatial smoothing extremes, namely global smoothness and independence. However, this flexibility leads to a large increase in the computational burden and a lack of parsimony causing problems of parameter identifiability, and a critique of the limitations of the existing literature in this area is given in Section 2. Here, we solve these problems with a novel prior elicitation method based on historical data, which is similar in spirit to power priors (see Chen and Ibrahim, 2006). Our elicitation is based on an approximate Gaussian likelihood, and produces a set of candidate correlation structures for the residual spatial autocorrelation. The LCAR prior combines a discrete uniform distribution on this set of candidate structures with a modified CAR prior for the random effects, which combined with the Poisson likelihood completes a full Bayesian hierarchical model. Inference is obtained using Markov chain Monte Carlo (MCMC) methods, and the model allows us to simultaneously estimate the random effects, their local spatial structure as well as the fixed effects. We conduct a large simulation study in Section 4 to show improved parameter estimation when using the proposed LCAR prior distribution. We follow up this investigation by analyzing the motivating data set for the city of Glasgow in Section 5. But first, we present the motivating data set and discuss the background modeling and prior distributions in Section 2.

## 2. Background

### 2.1. Motivating Study

**Figure 1:**
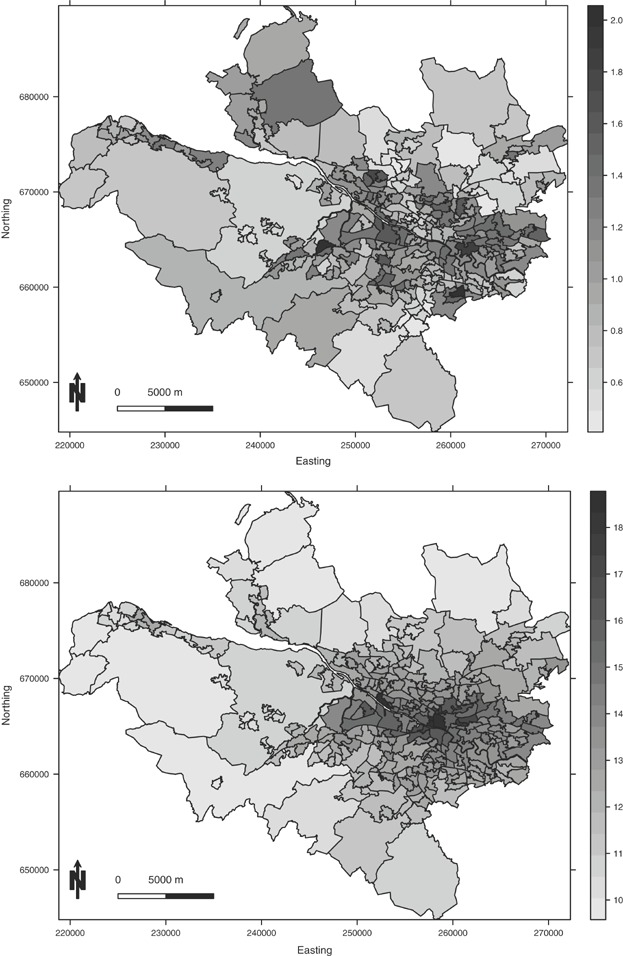
Maps displaying the spatial pattern in the standardized incidence ratio for respiratory disease in 2011 (top panel) and the modeled yearly average concentration (in μg m^−3^) of PM_10_ in 2010 (bottom panel).

The study region is the health board comprising the city of Glasgow and the river Clyde estuary, which in 2011 contained just under 1.2 million people. The region is partitioned into *n* = 271 administrative units called Intermediate Geographies (IG), which contain just over 4000 people on average. The data used in this study are freely available, and can be downloaded from the Scottish Neighbourhood Statistics (SNS) database (http://www.sns.gov.uk). The response variable is the numbers of admissions to non-psychiatric and non-obstetric hospitals in each IG in 2011 with a primary diagnosis of respiratory disease, which corresponds to codes J00-J99 and R09.1 of the International Classification of Disease tenth revision. Differences in the size and demographic structure of the populations living in each IG are accounted for by computing the expected numbers of hospital admissions using external standardization, based on age- and sex-specific respiratory disease rates for the whole study region. An exploratory estimate of disease risk is given by the standardized incidence ratio (SIR), which is the ratio of the observed to the expected numbers of admissions. It is displayed in the top panel of Figure1, and shows that the risks are highest in the heavily deprived east end of Glasgow (east of the study region) as well as along the southern bank of the river Clyde, the latter of which flows into the sea in the west and runs south east through the study region.

Ambient air pollution concentrations are measured at a network of locations across Scotland, details of which are available at http://www.scottishairquality.co.uk/. However, the network is not dense at the small-area scale required by this study, so instead we make use of modeled yearly average concentrations at a resolution of 1 km grid squares provided by the DEFRA (see http://laqm.defra.gov.uk/maps/). We use concentrations for 2010 in this study rather than 2011, because it ensures that the air pollution exposure occurred before the hospital admissions due to respiratory illnesses. These modeled concentrations were computed using dispersion models and were then calibrated against the available monitoring data, and further details are available from Grice et al. (2009). They were subsequently converted to the intermediate geography scale by computing the median value within each IG. Concentrations of carbon monoxide (CO, in mg m^−3^), nitrogen dioxide (NO_2_, in μg m^−3^), sulfur dioxide (SO_2_, in μg m^−3^) and PM are available for this study, the latter being measured as both PM_10_ (particles less than 10 μm in diameter) and PM_2.5_ (particles less than 2.5 μm in diameter). The PM_10_ data are displayed in the bottom panel of Figure1, which shows the highest concentrations are in the center of the city of Glasgow as expected.

A number of other covariates were considered in this study, the most important of which is a measure of socio-economic deprivation. The relationship between deprivation and ill health is well known (e.g., see Mackenbach et al., 1997), and in this study we use the percentage of people living in each IG in 2010 who are in receipt of job seekers allowance (JSA). Other variables we also consider are measures of ethnicity (the percentage of school children in each IG who are non-white), access to alternative forms of health care (the average time taken to drive to a doctor's surgery) and a measure of urbanicity (a factor variable with 6 levels, with level one defined as urban and level six as rural).

### 2.2. Modeling

The study region is partitioned into *n* areal units 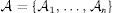, and the vectors of observed and expected numbers of disease cases are denoted by **Y** = (*Y*_1_, …, *Y*_*n*_) and **E** = (*E*_1_, …, *E*_*n*_, respectively. In addition, let 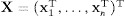 denote the matrix of *p* covariates and a column of ones for the intercept term, where the values relating to areal unit 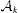 are denoted by 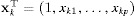. A Bayesian hierarchical model is typically used to model these data, and a general specification is given by (1)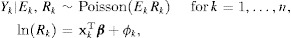 where the disease counts are assumed to be conditionally independent given the covariates and the random effects. Here, **β** = (*β*_0_, *β*_1_, …, *β*_*p*_ ) denotes the vector of covariate effects, while *R*_*k*_ represents disease risk in areal unit 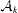. A value of *R*_*k*_ greater (less) than one indicates that areal unit 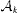 has a higher (lower) than average disease risk, and in terms of interpretation, *R*_*k*_ = 1.15 corresponds to a 15% increased risk of disease. As previously discussed, the random effects **ϕ** = (*ϕ*_1_, …, *ϕ*_*n*_) capture any residual spatial autocorrelation present in the disease data, and are typically assigned a CAR prior, which is a special case of a Gaussian Markov random field (GMRF). Such models are typically specified as a set of *n* univariate full conditional distributions, that is as *f*(*ϕ*_*k*_ | ***ϕ***_−*k*_ ) for *k* = 1, where ***ϕ***_−*k*_ = (*ϕ*_1_, …, *ϕ*_*k* − 1_, *ϕ*_*k* + 1_, …, *ϕ*_*n*_). However, the Markov nature of these models means that the conditioning is only on the random effects in geographically adjacent areal units, which induces spatial autocorrelation into ***ϕ***. The adjacency information comes from a binary *n* × *n* neighborhood matrix **W**, where *w*_*ki*_ equals one if areal units 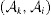 share a common border (denoted *k* ∼ *i*) and is zero otherwise (denoted *k* ≁ *i*). The intrinsic model (Besag et al., 1991; IAR) is the simplest prior in the CAR class, and its full conditional distributions are given by (2)
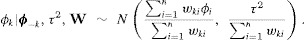


The conditional expectation is the mean of the random effects in neighboring areas, while the conditional variance is inversely proportional to the number of neighbors. The joint multivariate Gaussian distribution for ***ϕ*** corresponding to (2) has a mean of zero but a singular precision matrix **Q**(**W**)/τ^2^, where **Q**(**W**)= diag(**W1** – **W**), and **1** is an *n* dimensional vector of ones. This prior is appropriate if the residuals from the covariate component of the model, that is ln(**Y**/**E**) – **X*β***, are spatially smooth across the entire region, because the partial autocorrelation between (*ϕ*_*k*_, *ϕ*_*j*_) conditional on the remaining random effects (denoted ***ϕ***_−*kj*_ ) is (3)
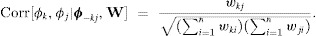


Equation (3) shows that all pairs of random effects relating to geographically adjacent areal units are partially autocorrelated (*w*_*kj*_ = 1), which smoothes the random effects across geographical borders. The most common extension to the IAR model to allow for varying levels of spatial smoothness is the BYM or convolution model (Besag et al., 1991), which augments the linear predictor in (1) with a second set of independent Gaussian random effects with a mean of zero and a constant variance. A further alternative using a single set of random effects was proposed by Stern and Cressie (1999), but this and other extensions have a single spatial autocorrelation parameter (for the BYM model it is the ratio of the two random effects variances) that controls the level of spatial smoothing globally across the entire region. Thus, these models are inappropriate for capturing the likely localized nature of the residual spatial autocorrelation, which may contain sub-region of spatial smoothness separated by step changes.

A small number of papers have extended the class of CAR priors to account for localized spatial smoothing, the majority of which have treated 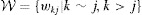 as a set of binary random quantities, rather than forcing them to equal one. The neighborhood matrix is always assumed to be symmetric so that changing *w*_*kj*_ also changes *w*_*jk*_, while the other elements in *W* relating to non-neighboring areal units remain fixed at zero. Equation (3) shows that this allows (*ϕ*_*k*_, *ϕ*_*j*_) corresponding to adjacent areal units to be conditionally independent or autocorrelated, and if *w*_*kj*_ (and hence *w*_*jk*_) is estimated as zero a boundary is said to exist between the two random effects. One of the first models in this vein was developed by Lu et al. (2007), who proposed a logistic regression model for the elements in 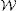, where the covariate was a non-negative measure of the dissimilarity between areal units 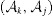. Similar approaches were proposed by Ma and Carlin (2007) and Ma, Carlin, and Banerjee (2010), who replace logistic regression with a second stage CAR prior and an Ising model, respectively. However, these approaches introduce a large number of partial autocorrelation parameters into the model, which for the Glasgow data considered here has *n* = 271 data points and 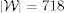 partial autocorrelation parameters. Therefore, full estimation of 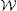 as a set of separate unknown parameters results in a highly overparameterized precision matrix for ***ϕ***, and Li, Banerjee, and McBean (2011) suggest that the individual elements are poorly identified from the data and are computationally expensive to update.

A related approach was proposed by Lee and Mitchell (2012), who deterministically model the elements of 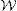 as a function of measures of dissimilarity and a small number of parameters, rather than modeling each element as a separate random variable. However, their approach is designed for the related fields of disease mapping and Wombling, whose aims are not, as they are here, to estimate the effects of an exposure on a response. An alternative approach was suggested by Lee and Mitchell (2013), who propose an iterative algorithm in which 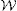 is updated deterministically based on the joint posterior distribution of the remaining model parameters. However, their algorithm has the drawback that only an estimate of each *w*_*kj*_ is provided, rather than the posterior probability that *w*_*kj*_ = 1. Finally, Reich et al. (2006) and Hughes and Haran (2013) take an alternative approach, and force the random effects to be orthogonal to the covariates using a residual projection matrix.

## 3. Methodology

Our methodological approach follows the majority of the literature critiqued above, and treats the elements in 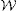 relating to contiguous areal units as a set of binary random quantities. As CAR priors are a special case of an undirected graphical model, we follow the terminology in that literature and refer to 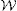 as the set of *edges*, and further define any edge 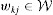 that is estimated as zero as being removed. Our methodological innovation is a LCAR prior, which comprises a joint distribution for an extended set of random effects 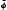 and the set of edges 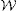, rather than the traditional approach of assuming the latter is fixed. We decompose this joint prior distribution as 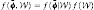, and the next three sub-sections describe its two components as well as the overall hierarchical model.

### 3.1. Prior Distribution ’ 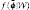


The IAR prior given by (2) is an inappropriate model for ***ϕ*** in the context of treating 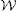 as random, because of the possibility that all of the edges for a single areal unit could be removed. In this case, 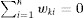 for some *k*, resulting in (2) having an infinite mean and variance. Therefore we consider an extended vector of random effects 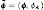, where ϕ_*_ is a global random effect that is potentially common to all areal units and prevents any unit from having no edges. The extended (*n* + 1) × (*n* + 1) dimensional neighborhood matrix corresponding to 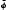 is given by (4)
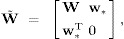
 where W_*_ = (*w*_1*_, …, *w*_n*_) and 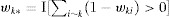. Here, I[.] denotes an indicator function, so that *w*_k*_ = 1 if at least one edge relating to areal unit 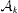 has been removed, otherwise *w*_k*_ equals zero. Based on this extended neighborhood matrix, we propose modeling 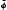 as 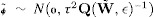, where the precision matrix is given by (5)
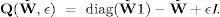


The component 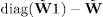 corresponds to the IAR model applied to the extended random effects vector 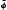, while the addition of *ϵI* ensures the precision matrix is diagonally dominant and hence invertible. The requirement for 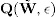 to be invertible comes from the need to calculate its determinant when updating 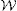, a difficulty not faced when implementing model (2) because 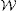 and hence **Q**(**W**) are fixed. The addition of a small positive constant *ϵ* to the diagonal of the precision matrix has been suggested in this context by Lu et al. (2007). A sensitivity analysis to different values of *ϵ* was conducted in the simulation study in Section 4, and the results were robust to this specification. Therefore, we recommend setting *ϵ* = 0.0001 when implementing the model. The full conditional distributions corresponding to the LCAR model are given by: (6)
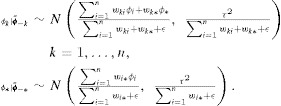


In (6), the conditional expectation is a weighted average of the global random effect ϕ_*_ and the random effects in neighboring areas, with the binary weights depending on the current value of 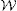. This shows that ϕ_*_ acts as a global non-spatial random effect, which influences the conditional expectation of any other random effect that corresponds to an areal unit with at least one edge removed. The conditional variance is approximately (due to *ϵ*) inversely proportional to the number of edges remaining in the model, including the edge to the global random effect ϕ_*_. Removing the *kj*th edge from 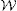 sets *w*_*kj*_ (and hence *w*_*jk*_) equal to zero and makes (*ϕ*_*k*_, *ϕ*_*j*_) conditionally independent, and means that the global random effect ϕ_*_ is included in the conditional expectation to allow for non-spatial smoothing. In the extreme case of all edges being retained in the model (6) simplifies to the IAR model for global spatial smoothing, while if all edges are removed the random effects are independent with a constant mean and variance, which are approximately (again due to *ϵ*) equal to ϕ_*_ and τ^2^, respectively.

### 3.2. Prior Distribution –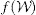


The dimensionality of 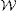 is 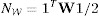, and as each edge is binary the sample space has size 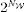. The simplest approach would be to assign each edge an independent Bernoulli prior, but as described in Section two this is likely to result in 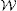 being weakly identifiable. Therefore we treat 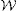 as a single random quantity, and propose the following discrete uniform prior for its neighborhood matrix representation 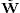; (7)
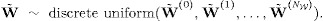


The last candidate value 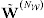 retains all 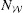 edges in the model, that is 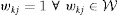, and corresponds to the IAR model for global spatial smoothing. Moving from 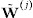 to 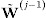 removes an edge from 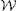, which sets one additional *w*_*kj*_ = *w*_*jk*_ = 0. This means that 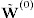 contains no edges and corresponds to independent random effects. Thus, the set 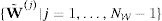 corresponds to localized spatial smoothing, where some edges are present in the model and the corresponding random effects are smoothed, while other edges are absent and no such smoothing is enforced. This restriction reduces the sample space of 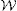 to being one-dimensional, because the possible values 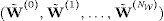 have a natural ordering in terms of the number of edges present in the model.

We propose eliciting the set of candidate values 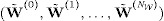 from disease data prior to the study period, because such data are typically available and should have a similar spatial structure to the response. Let 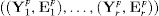 denote these vectors of observed and expected disease counts for the *r* time periods prior to the study period. The general likelihood model (1) gives the vector of expectations for the study data as 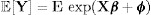, which is equivalent to 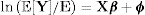. Then as 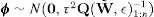, we make the approximation (8)
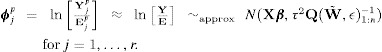


Based on this approximation, the prior elicitation takes the form of an iterative algorithm, which begins at 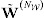 (which retains all edges in the model) and moves from 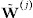 to 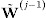 by removing a single edge from 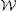. The algorithm continues until it reaches 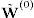, where all edges have been removed. The algorithm moves from 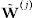 to 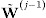 by computing the joint approximate Gaussian log-likelihood for 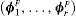 based on (8). This is given by (9)
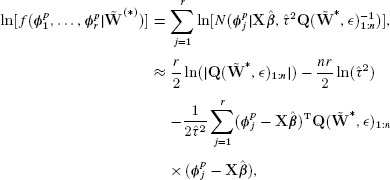
 where the constant in the likelihood function has been removed. This likelihood approximation is calculated for all matrices 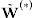 that differ from 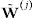 by having one additional edge removed. From this set of candidates, 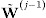 is equal to the value of 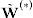 that maximizes the above log-likelihood. This prior elicitation approach removes edges from 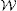 in sequence conditional on the current value of 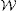, rather than naively treating each edge independently of the others. However, this approach requires (9) to be evaluated 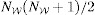 times, which makes the approach computationally intensive. This computational burden is reduced by estimating 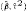 by maximum likelihood, that is, based on 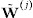, 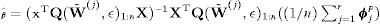 and 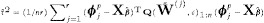. In addition, to speed up the computation of the quadratic form in (9), the above estimators are based on 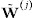 rather than on each individual 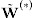.

### 3.3. Overall Model

The Bayesian hierarchical model proposed here combines the likelihood (1) with the priors (6) and (7) and is given by (10)
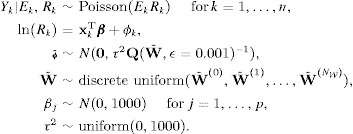


Diffuse priors are specified for the regression parameters ***β*** and the variance parameter τ^2^, while *ϵ* is set equal to 0.001. A sensitivity analysis to the latter is presented in Section 4, which shows that model performance is not sensitive to this choice. Inference for this model is based on MCMC simulation, using a combination of Metropolis–Hastings and Gibbs sampling steps. The spatial structure matrix 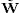 is updated using a Metropolis–Hastings step, where if the current value in the Markov chain is 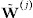, then a new value is proposed uniformly from the set 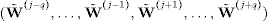. Here *q* is a tuning parameter, which controls the mixing and acceptance rates of the update. Functions to implement model (10) as well the prior elicitation are available in the statistical software R, and are provided in the Supplementary Material accompanying this article. The increased flexibility provided by the LCAR model inevitably means that it is more computationally demanding than the commonly used BYM model. Specifically, it takes 90% longer to produce the same number of MCMC samples compared with the BYM model, while the prior elicitation step takes around 40 s for the Glasgow data considered here.

## 4. Simulation Study

This section presents a simulation study, which compares the performance of the LCAR model proposed here against the BYM model and the recent innovations proposed by Lee and Mitchell (2013) for localized spatial smoothing (hereafter referred to as LM) and Hughes and Haran (2013) for smoothing orthogonal to the covariates (hereafter referred to as HH). For the latter, *q* = 50 basis functions are used, because it is the default choice in the *ngspatial* software. However, we applied the model with a range of different *q* values, and the results showed little sensitivity to this value.

**Figure 2:**
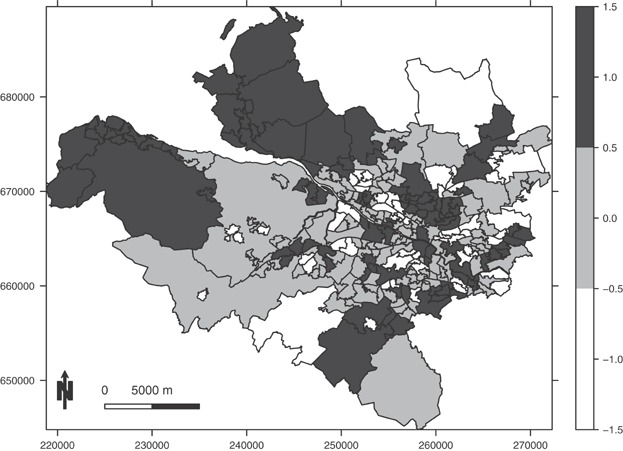
A map showing the piecewise constant mean function (with possible values {−1, 0, 1}) for the random effects that generate localized spatial correlation in the simulation study.

### 4.1. Data Generation and Study Design

Simulated data are generated for the 271 IGs that comprise the Greater Glasgow study region described in Section 2. Disease counts are generated from model (1), where the size of the expected numbers **E** is varied to assess its impact on model performance. The log risk surface is a linear combination of a single spatially smooth covariate acting as air pollution, and localized residual spatial autocorrelation. The pollution covariate is generated as the average of two Gaussian spatial processes with different ranges, one of which has the same range and hence is confounded with the localized spatial autocorrelation. Both spatial processes are generated using the Matérn family of correlation functions, where the smoothness parameter equals 2.5. The regression coefficient for the covariate is fixed at *β* = 0.1, while new realizations of the covariate and the residual spatial autocorrelation are generated for each simulated data set. The residual autocorrelation is also generated from a Gaussian process with a Matérn correlation function, where localized spatial structure is induced via a piecewise constant mean. The template for this is shown in Figure2, and only has three distinct values {−1, 0, 1}. These values are multiplied by a constant *M* to obtain the expectation, where larger values of *M* lead to bigger step changes in the spatial surface. The study is split into nine different scenarios comprising pairwise combinations of *M* = 0.5, 1, 1.5 and *E*_*k*_ ϵ [10, 25], [50, 100] and [150, 200]. The size of **E** quantifies disease prevalence, while *M* determines the extent of local rather than global residual autocorrelation (larger values correspond to more prominent localized structure). Each simulated data set consists of study data and 3 years of prior data, which is the number of prior data sets used in the Glasgow motivating study. The residual spatial autocorrelation for the latter is generated by adding uniform random noise in the range [−0.1, 0.1] to the realization generated for the real data, which mimics the realistic situation where the spatial patterns in the prior and real data are similar but not identical.

### 4.2. Results

**Figure 3:**
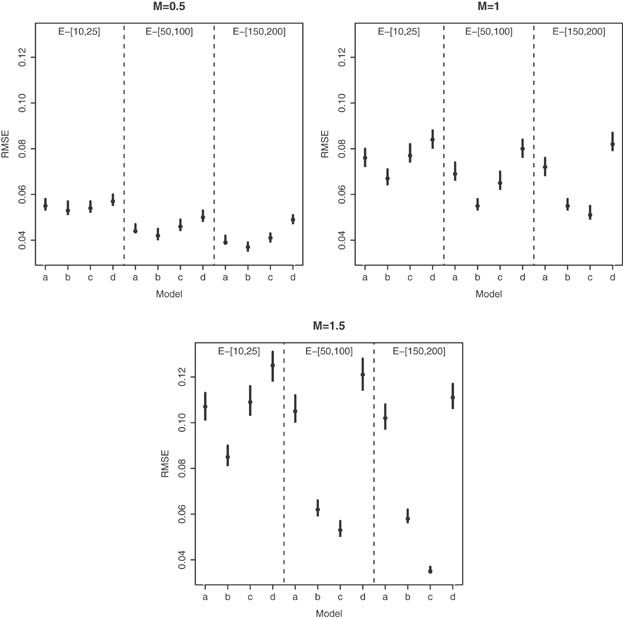
Root mean square errors (RMSE) for the estimated regression parameter *β*. In each case, the dot represents the estimated RMSE while the black bars are bootstrapped 95% uncertainty intervals. The models are: (a) BYM, (b) LCAR, (c) the model of Lee and Mitchell (2013), and (d) the model of Hughes and Haran (2013).

Five hundred data sets are generated under each of the nine scenarios and the results are displayed in Figure3 and Table 1, which, respectively, summarize the root mean square error (RMSE) of the estimated regression parameter and the coverage and widths of the 95% uncertainty intervals. The back dots in the figure display the RMSE values for all four models, while the vertical lines represents bootstrapped 95% uncertainty intervals based on 1000 bootstrapped samples. The figure shows that no single model exhibits the lowest RMSE values for all scenarios, as the LCAR model performs best in this regard for six scenarios and second best in the remaining three, while the LM model has the lowest values for three scenarios. The latter performs well when the magnitude of the localized structure is large (large *M*), which is likely to be because it induces localized smoothness only when there are substantial differences between neighboring random effects. In contrast, it performs on a par with the BYM model when the localized structure is less prominent, and is substantially worse than the LCAR model in these situations. The HH model performs consistently poorly relative to the other models, which is likely to be because although it induces spatial smoothing orthogonal to the covariates, the smoothing is global (each basis function is a globally smooth quantity) and does not allow adjacent areas to have very different values (step changes). The figure also illustrates the importance of choosing an appropriate model for spatial autocorrelation, as reductions in RMSE between the best and worst model range between 6.3% and 68.3% depending on the scenario. The differences between the models can also be substantial, as the bootstrapped 95% uncertainty intervals for the RMSE often do not overlap.

**Table 1:** Percentage coverages and average widths (in brackets) for the 95% credible intervals for the estimated regression parameter *β*. Here LM and HH refer to the models proposed by Lee and Mitchell (2013) and Hughes and Haran (2013)

		Model			
		
E	*M*	BYM	LCAR	LM	HH
	0.5	94.2 (0.204)	92.2 (0.193)	92.8 (0.197)	73.8 (0.131)
[10, 25]	1	94.2 (0.290)	92.8 (0.248)	91.0 (0.266)	53.0 (0.128)
	1.5	94.4 (0.392)	93.0 (0.298)	80.0 (0.284)	32.8 (0.122)
	0.5	92.6 (0.158)	90.2 (0.134)	86.6 (0.139)	46.2 (0.065)
[50, 100]	1	94.0 (0.257)	89.8 (0.184)	73.8 (0.148)	28.0 (0.063)
	1.5	90.8 (0.365)	92.8 (0.236)	79.0 (0.134)	20.4 (0.060)
	0.5	94.2 (0.147)	89.6 (0.113)	78.2 (0.099)	31.4 (0.042)
[150, 200]	1	90.2 (0.248)	85.8 (0.165)	67.0 (0.098)	18.0 (0.041)
	1.5	92.4 (0.353)	93.0 (0.218)	81.4 (0.087)	12.6 (0.040)

Table 1 shows that overall the uncertainty intervals from the BYM model are closest to their nominal 95% coverage levels, with values above 90% for all scenarios. The intervals from the LCAR model are also close to their nominal levels in most scenarios, with all but three being above 90%. However, the generally small increases in coverages exhibited by the BYM model compared to the LCAR model come at the cost of wider uncertainty intervals, which are between 5.4% and 38.2% wider depending on the scenario. The coverages from the LM model are relatively poor in comparison, being between 67.0% and 92.8%, respectively. Finally, the intervals from the HH model exhibit very poor coverage, which is likely to be due to both the relatively poor estimation performance as summarized by Figure3 and their comparatively narrow average widths. RMSE values for the fitted values *E*_*k*_*R*_*k*_ and coverage probabilities for the corresponding uncertainty intervals are displayed in the Supplementary Material accompanying this article, and show broadly similar but less dramatic patterns to the results presented here for the fixed effects. Finally, a sensitivity analysis to the choice of the diagonally dominant constant *ϵ* was conducted, where the middle values of *M* = 1 and *E*_*k*_ *ϵ* [50, 100] were used. Values of *ϵ* = 0.0001, 0.001, and 0.01 were considered, and the results were robust to this choice.

**Table 2:** A summary of the overall fit of each model (top panel) and the estimated covariate effects (bottom panel)

	Model			
	
	BYM	LCAR	LM	HH
DIC	2124.0 (178.5)	2112.4 (173.4)	2115.8 (167.7)	2467.6 (157.1)
Moran's I	−0.025 (0.7078)	−0.082 (0.9834)	−0.121 (0.9997)	−0.089 (0.9909)
JSA	1.304 (1.268, 1.342)	1.283 (1.247, 1.320)	1.306 (1.272, 1.341)	1.318 (1.300, 1.336)
CO	0.997 (0.954, 1.038)	1.011 (0.973, 1.045)	0.998 (0.959, 1.036)	1.021 (1.006, 1.035)
NO_2_	1.036 (0.998, 1.072)	1.040 (1.012, 1.067)	1.033 (1.003, 1.065)	1.043 (1.028, 1.059)
PM_2.5_	1.029 (0.991, 1.067)	1.039 (1.007, 1.071)	1.026 (0.989, 1.063)	1.035 (1.021, 1.050)
PM_10_	1.032 (0.994, 1.071)	1.040 (1.007, 1.073)	1.028 (0.993, 1.064)	1.034 (1.021, 1.048)
SO_2_	1.009 (0.980, 1.040)	1.016 (0.989, 1.044)	1.010 (0.983, 1.037)	1.010 (0.998, 1.024)

The former includes the DIC (effective number of parameters in brackets) and the Moran's I statistic applied to the residuals (*p*-value in brackets). The estimated covariate effects are presented as relative risks for a one standard deviation increase in each covariates value, which are JSA (2.78%), CO (0.0076 mg m^−3^), NO_2_ (5.0 **μ**g m^−3^), PM_2.5_ (1.1**μ**g m^−3^), PM_10_ (1.5**μ** g m^−3^), and SO_2_ (0.48**μ**g m^−3^). Here, LM and HH refer to the models proposed by Lee and Mitchell (2013) and Hughes and Haran (2013).

## 5. Results from the Glasgow Study

### 5.1. Modeling

Initially, a simple Poisson log-linear model including the four non-pollution covariates was fitted to the data, and only JSA exhibited a significant relationship with respiratory disease risk. The remaining three covariates were thus removed from the model, and each of the pollutants were included in separate models due to their collinearity. The residuals from these models exhibited substantial overdispersion, with an estimate of 3.47 when PM_2.5_ was included in the model. The presence of residual spatial autocorrelation was assessed by a permutation test based on Moran's I statistic, which yielded a highly significant *p*-value of 0.00001. Random effects were thus added to the model, and we implement the four models compared in the simulation study. These models induce different types of spatial smoothing, and include the commonly used BYM model for global spatial smoothing, the LCAR model proposed here and the proposal of Lee and Mitchell (2013) for localized spatial smoothing, and the model proposed by Hughes and Haran (2013) for smoothing orthogonal to the covariates. Finally, for the LCAR model the prior elicitation was based on respiratory disease data from 2008 to 2010.

### 5.2. Results –Model Fit

Posterior inference for all models was based on three parallel Markov chains, with the exception being the model proposed by Lee and Mitchell (2013), which uses integrated nested Laplace approximations (INLA) instead of MCMC simulation. These chains were burnt in for a period of 50,000 iterations, by which time convergence was assessed to have been reached, and then run for an additional 50,000 iterations, yielding 150,000 samples in total. The results are displayed in Table 2, which quantifies the overall goodness-of-fit of the models and the estimated covariate effects. The results relating to model fit are those where PM_2.5_ was the pollution metric, but similar results were obtained for the other pollutants. The goodness-of-fit of each model is summarized by its deviance information criterion (DIC; Spiegelhalter et al., 2002), where a smaller value represents a better fitting model. The table shows that the LCAR model exhibits the best fit to the data according to the DIC, while the LM model is the next best in that regard. In particular, both these localized smoothing models appear to fit the data better than the global smoothing BYM model, with differences of 11.6 and 8.2, respectively. The HH model exhibits the worst fit to the data in terms of DIC, which is likely to be because it contains *q* = 50 basis functions compared with the 271 random effects used by the other models. The presence of residual spatial autocorrelation was then assessed using a Moran's I permutation test (based on 10,000 random permutations), and all four models had removed the spatial autocorrelation present in the residuals from the covariate only model.

### 5.3. Results –Covariate Effects

Table 2 also displays the estimated relationships between each covariate and the response, where all results are presented as relative risks for an increase of one standard deviation in each covariates value. The table shows that NO_2_ and both PM metrics exhibit substantial effects on respiratory disease risk, as their estimated relative risks range from 1.026 to 1.043 depending on the pollutant chosen and the model that was fitted. In contrast, neither CO nor SO_2_ exhibit any substantial health impact, as both have relative risks close to the null risk of one for the majority of the four models. The estimated relative risks for a single pollutant show considerable variation between the four models, which suggests that the choice of spatial smoothing prior impacts on the fixed effects estimates. This result thus confirms the results of Reich et al. (2006), and the simulation study conducted here suggests that the estimates from the LCAR model are likely to be the most accurate. Based on those results NO_2_, PM_2.5_, and PM_10_ exhibit substantial effects on respiratory ill health (95% credible intervals do not include the null risk of one), with relative risks of 4.0%, 3.9%, and 4.0%, respectively. Consistent attenuation of the estimated pollution effects are observed for the global smoothing BYM model (and the LM model) compared with the LCAR and HH models, which may be due to the collinearity between the fixed and random effects. Also noteworthy is the substantially smaller credible intervals obtained from the HH model compared with those from the other models, which is consistent with the simulation study results. Finally, we note that while the risks estimated in this study are relatively large, they are broadly in line with existing studies such as Lee et al. (2009) and Haining et al. (2010). Furthermore, these risks should not be compared with those estimated from short-term time series studies, because a fixed μgm^−3^ increase in a persons short-term exposure is likely to have a smaller health impact than the same increase in their exposure over the long-term.

### 5.4. Results –Localized Residual Spatial Autocorrelation

**Figure 4:**
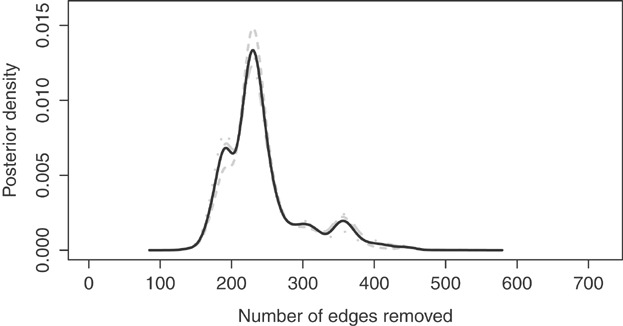
Posterior density for the number of edges removed from the model. The three grey lines display the estimates from the individual Markov chains, while the bold black line displays the combined density from all three chains.

Figure4 displays the posterior distribution for the number of edges removed from the model, where the three grey lines are chain specific estimates while the bold black line represents the combined distribution from all three Markov chains. The figure shows close agreement between the chains, as all three give similar density estimates. There are 718 edges in total in the Greater Glasgow region, and the middle 95% of the posterior distribution lies between 171 and 385 of these having been removed. The figure suggests that while the posterior variability is relatively wide, there is information in the data to estimate the number of edges to remove. Specifically, the posterior distribution is multi-modal, with the largest mode occurring when 231 edges are removed. The figure also provides strong evidence that the random effects are neither globally spatially smooth not independent, as there is no posterior mass at either end of the range of possible values (0 or 718 edges removed).

## 6. Discussion

This article has proposed a new LCAR prior for modeling residual spatial autocorrelation, which is flexible enough to capture either spatial smoothness or a distinct step change in the data between adjacent areal units. This flexibility is due to the treatment of the neighborhood matrix **W** as a random quantity, rather than assuming it is fixed based on geographical adjacency. However, this requires a large number of partial correlation parameters to be estimated, and the resulting lack of parsimony is overcome by using prior information to greatly reduce the size and dimensionality of the sample space for **W**. The proposed model can estimate a range of localized spatial autocorrelation structures, as well as patterns that are globally smooth or independent in space. These residual autocorrelation structures are also unlikely to be collinear to the fixed effects, because they are elicited from the prior information after the covariate effects have been removed.

The simulation study has shown that the LCAR model exhibits generally superior estimation performance for fixed effects compared with both the commonly used BYM model and the recent innovations by Lee and Mitchell (2013) and Hughes and Haran (2013). It generally estimated the fixed effects with the smallest RMSE, had coverages only slightly below their nominal levels, and had narrower credible intervals than the BYM model. This superior performance is likely to result from the LCAR model having the flexibility to represent a range of localized spatial autocorrelation structures, which by construction are unlikely to be collinear to the estimated fixed effects. In this sense, it contains the localized spatial smoothing aspects of Lee and Mitchell (2013), while having a high likelihood of not producing random effects that are collinear to the fixed effects as in Hughes and Haran (2013). The final conclusion from the simulation study is that inappropriate control for residual spatial autocorrelation can greatly retard fixed effects estimation, meaning that its careful modeling is vital even if it is not itself of direct interest.

The epidemiological study presented in this article shows substantial evidence that particulate air pollution and NO_2_ are harmful to respiratory health in Greater Glasgow, with an estimated increase in the population's disease burden of around 4% if yearly average concentrations increased by one standard deviation. However, one must remember that this is an observational ecological study design, and the results must not be interpreted in terms of individual level cause and effect (ecological bias). Even so, as small-area studies are cheaper and quicker to implement than individual level cohort studies, they form an important component of the evidence base quantifying the health effects of long-term exposure to air pollution.

There are many avenues for future work in this area, including the extension of the methodology to the spatio-temporal domain. In an epidemiological context, the extension of the present study to the whole of the United Kingdom would be of interest to policymakers, as it would give the UK government a national rather than a regional picture of the extent of the air pollution problem. In addition, while the motivation for this article was an ecological regression problem, the methodology developed will also be directly relevant to the fields of disease mapping and Wombling, whose aims are to estimate the spatial pattern in disease risk and to identify any boundaries in the estimated risk surface.

## 7. Supplementary Materials

This paper contains on-line supplementary material including additional simulation results, software (functions in R) to implement the LCAR model, the data used in the Glasgow air pollution study, and code to partially re-create the analysis presented in Section 5. These materials are available with this paper at the *Biometrics* website on Wiley Online Library.

## Supporting Information

Supporting Information.

Supplementary Materials Code.
