# Supplementary material for: A Bayesian localized conditional autoregressive model for estimating the health effects of air pollution
Source: Biometrics. 2014 Feb 24;70(2):419–29. doi: 10.1111/biom.12156 (PMC4282098; doi:10.1111/biom.12156)

Web-based Supplementary Materials for 'A Bayesian localised conditional autoregressive model for estimating the health effects of air pollution' by Duncan Lee, Alastair Rushworth and Sujit K. Sahu

Web Appendix A

The supplmentary material to this paper consists of two parts. The first is computer software and data, which allows the data analysis in section 5 to be partially reproduced. The data are stored in *data.csv* which contains the repsonse and covariate data, and *Wadjacency.txt* which contains the binary neighbourhood matrix for the Greater Glasgow sutdy region. The R functions *prior.elicit.LCAR.R* and *poisson.discreteWLCAR.R* carry out the prior elicitation of the set of candidate $\mathbf{W}$ matrices and run the LCAR model respectively. Finally, the R file *Run the Greater Glasgow study.R* gives code to partially reproduce the data analysis in section 5. The second part of the supplementary material is additional simulation study results, which are presened below.

Web Appendix B

Figure 1 shows the estimated root mean square errors (RMSE) for the fitted values E_kR_k for all models and scenarios, and is in the same format as the corresponding figure from the main paper. The main finding is that the BYM, LCAR and LM models exhibit similar RMSE values for all scenarios, where as the HH model does substantially worse. This result is not surprising, as the HH model is designed for fixed efffect estimation and is much more parsimonious (50 basis functions compared with 271 random effects) than the other three models. The relative performances of the BYM, LCAR and LM models are similar for all scenarios, with differences between them ranging between 0.21 and 1.39 on the scale of Figure 1. In addition, we note that the bootstrapped uncertainty intervals are very small for the fitted values, which is why they cannot be seen on the scale used in the graph. Finally, the coverage probabilities of the 95% credible intervals for the fitted values are summarised in Table 1. The table

Web Table 1: Percentage coverages for the 95% credible intervals for the fitted values E_kR_k. Here LM and HH refer to the models proposed by Lee and Mitchell (2013) and Hughes and Haran (2013).

**E**	M	**Model**			
		**BYM**	**LCAR**	**LM**	**HH**
	0.5	93.7	95.6	94.0	61.7
$[10, 25]$	1	94.4	96.2	92.1	46.1
	1.5	94.8	96.1	90.1	34.0
	0.5	94.7	96.0	92.1	43.0
$[50, 100]$	1	95.0	95.9	89.5	26.5
	1.5	94.8	95.5	89.4	17.8
	0.5	94.8	95.6	90.4	31.4
$[150, 200]$	1	94.9	95.5	89.9	17.9
	1.5	95.0	95.3	90.7	11.9

shows that the BYM and LCAR models attain almost their nominal coverage levels, whereas those from the LM model are slightly low. In contrast, those from the HH model are very low, which is due to the relatively poor estimation performance as a result of the relative parsimony of this model compared with the others.

Web Figure 1: Root mean square errors (RMSE) for the fitted values $E_k R_k$. The models are: a - BYM, b - LCAR, c - the model of Lee and Mitchell (2013), and d - the model of Hughes and Haran (2013).

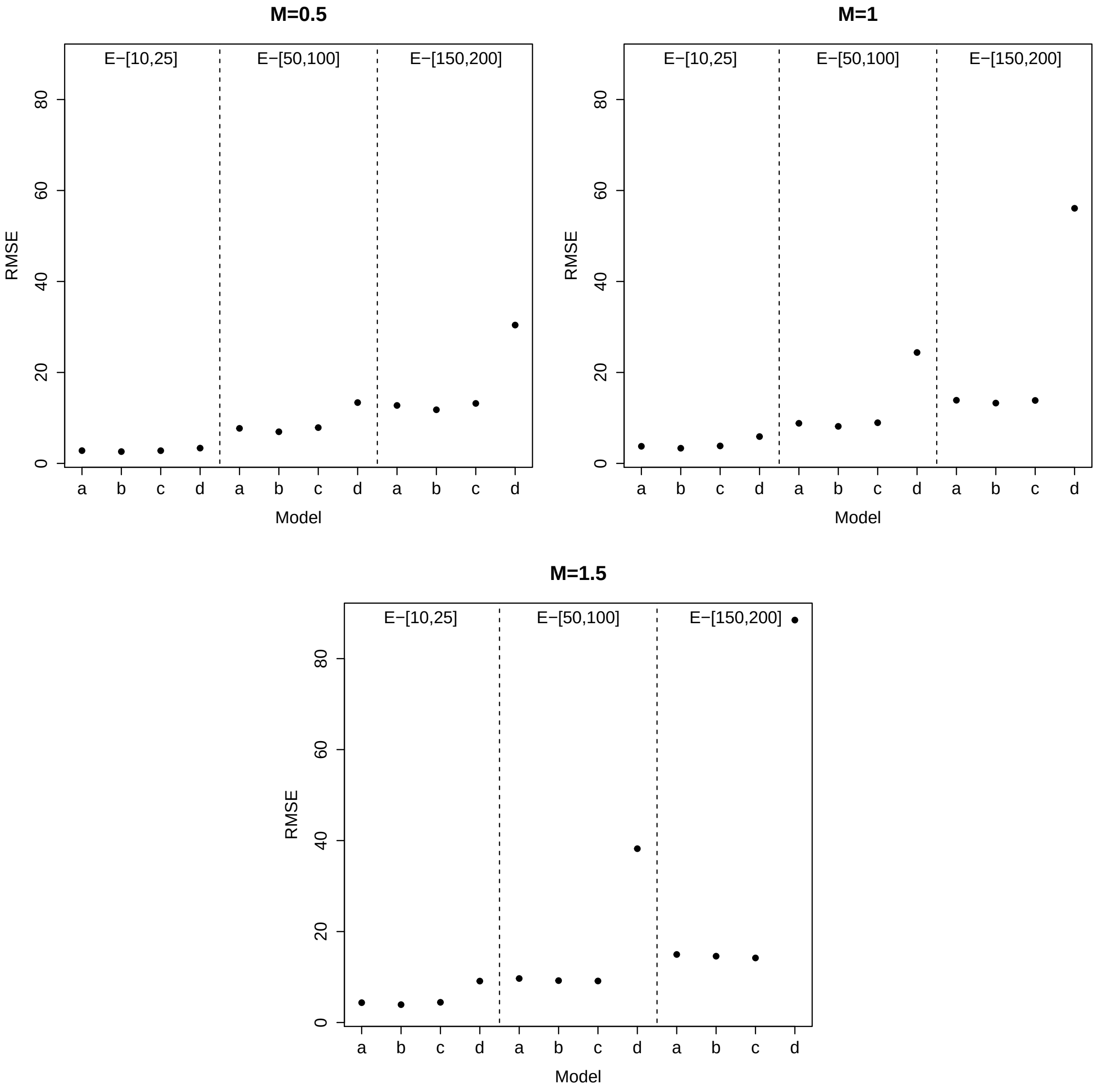

Supplement: Supplementary file 2 — Supporting Information. [file biom0070-0419-SD2.pdf]
